# Programmed Death 1 Deficiency Induces the Polarization of Macrophages/Microglia to the M1 Phenotype After Spinal Cord Injury in Mice

**DOI:** 10.1007/s13311-013-0254-x

**Published:** 2014-05-23

**Authors:** Anhui Yao, Fangfang Liu, Kun Chen, Liang Tang, Ling Liu, Kun Zhang, Caiyong Yu, Ganlan Bian, Hongmin Guo, Jingjing Zheng, Peng Cheng, Gong Ju, Jian Wang

**Affiliations:** 1 Institute of Neurosciences, the Fourth Military Medical University, 169 West Changle Road, Xi’an, 710032 China; 2 The 153 Hospital of People’s Liberation Army, Zhengzhou, China; 3 The 150 Hospital of People’s Liberation Army, Luoyang, China

**Keywords:** Macrophage, Microglia, Polarization, Programmed death 1, Spinal cord injury

## Abstract

The inflammatory response following spinal cord injury (SCI) involves the activation of resident microglia and the infiltration of macrophages. Macrophages and microglia can be polarized into the classically activated proinflammatory M1 phenotype or the alternatively activated anti-inflammatory M2 phenotype. Programmed cell death 1 (PD-1) is a critical immune inhibitory receptor involved in innate and adaptive immune responses. However, whether PD-1 is involved in the modulation of macrophage/microglial polarization is unknown. In this study, the mRNA levels of *pd1* gradually increased after SCI, and PD-1 protein was found in macrophages/microglia in injured spinal cord sections. PD-1 knockout (KO) mice showed poor locomotor recovery after spinal cord crushing compared with wild-type mice. M1-type macrophages/microglia accumulated in greater numbers in the injured spinal cord of PD-1-KO mice. Under polarized stimulation, induced expression of PD-1 occurred in cultured macrophages and microglia. PD-1 suppressed M1 polarization by reducing the phosphorylation of signal transducer and activator of transcription 1 (STAT1) and promoted M2 polarization by increasing STAT6 phosphorylation. In PD-1-KO mice, the M1 response was enhanced via the activation of STAT1 and nuclear factor-kappa B. Furthermore, PD-1 played various roles in phagocytosis in macrophages and microglia. Therefore, our results suggest that PD-1 signaling plays an important role in the regulation of macrophage/microglial polarization. Thus, deregulated PD-1 signaling may induce the polarization of macrophages/microglia toward the M1 phenotype. Overall, our results provide new insights into the modulatory mechanisms of macrophage/microglial polarization, thereby possibly facilitating the development of new therapies for SCI via the regulation of macrophage/microglial polarization through PD-1 signaling.

**Electronic supplementary material:**

The online version of this article (doi:10.1007/s13311-013-0254-x) contains supplementary material, which is available to authorized users.

## Introduction

The inflammatory response plays an important role in the pathogenesis of spinal cord injury (SCI) [1, 2]. Macrophages/microglia are the predominant inflammatory cells responsible for this response, and can be polarized into the “classically activated” M1 phenotype or the “alternatively activated” M2 phenotype depending on the signals within the lesion microenvironment [3]. Under *in vitro* conditions, exposure to lipopolysaccharide (LPS) and interferon gamma (IFN-γ) activates M1 macrophages, whereas the presence of interleukin 4 (IL-4) or IL-13 activates the M2 phenotype [4]. M1 macrophages/microglia produce high levels of proinflammatory cytokines, such as IL-12, IL-1β, and tumor necrosis factor alpha, in addition to increased levels of oxidative metabolites, such as inducible nitric oxide synthase (iNOS) [5, 6]. These inflammatory factors are promoted by the IFN-γ-mediated signal transducer and activator of transcription 1 (STAT1) signaling pathway. In contrast, M2 macrophages produce high levels of arginase 1 (Arg1), mannose receptor (CD206), IL-10, transforming growth factor beta (TGF-β), and several neurotrophic factors, such as ciliary neurotrophic factor, insulin-like growth factor, epidermal growth factor, and nerve growth factor, all of which suppress inflammatory responses and facilitate axonal regeneration. These molecules are mediated by the STAT6 signaling pathway [3]. In SCI, 2 distinct macrophage/microglia subsets (M1 or M2) with opposing effects (neurotoxicity or regeneration) have been identified based on their ratio during pathological processes [7]. In the first few days after SCI, macrophages/microglia upregulate the levels of iNOS and proinflammatory cytokines (TNF-α, IL-1β, and IL-6), which polarize and maintain these cells toward the M1 phenotype [8, 9]. Kigerl et al. [7] reported that M1 macrophages/microglia are maintained in lesion sites weeks after injury in the contused mouse spinal cord; however, the number of M1 cells is markedly reduced 14 and 28 days after SCI. Furthermore, inhibitory signals may prevent the overactivation of M1 macrophages/microglia after SCI [10–12].

Programmed death 1 (PD-1 or CD279) is a 288-amino acid type-I transmembrane protein that belongs to the CD28 superfamily and shares 23 % amino acid sequence homology with cytotoxic T-lymphocyte-associated antigen 4 [11, 12]. As a co-inhibitory receptor, PD-1 and its ligands—PD-L1 (B7-H1; CD274) and PD-L2 (B7-DC; CD273)—elicit inhibitory signals that regulate the balance between T-cell activation, tolerance, and immune-mediated tissue damage [11, 12]. The expression of PD-1 on T cells, natural killer cells, B cells, and monocytes is induced upon activation; thus, PD-1 plays critical roles in the regulation of autoimmunity, tumor immunity, viral/parasite immunity, the innate inflammatory response, and allergy, and in immune privilege [11–13]. The binding of PD-L1 to PD-1 recruits *src* homology region 2 domain-containing phosphatase 2 (SHP-2) or SHP-1/SHP-2 with the cytoplasmic domain immunoreceptor tyrosine-based inhibitory motif (ITIM) and immunoreceptor tyrosine-based switch motif, both of which inhibit proliferation and cytokine production in T lymphocytes [11]. Although the role of PD-1 in the adaptive immune system has been well characterized [10–12], its function in the innate immune system has only recently been investigated. Several studies have revealed a possible role for PD-1 in macrophages [13–17]; however, whether PD-1 is involved in the regulation of the polarization of macrophages is still unknown. In this study, we show that PD-1 deficiency promotes M1 polarization of macrophages/microglia and exacerbates locomotor recovery after SCI. We also demonstrate that PD-1 deficiency *in vitro* regulates the M1/M2 polarization of macrophages/microglia via STAT1 or STAT6 phosphorylation, thereby playing opposing roles for phagocytosis in macrophages and microglia.

## Materials and Methods

### Animals

PD-1 knockout (KO) (*Pdcd*-*1* ^-/-^) male mice (a gift from Dr. Tasuku Honjo, Kyoto University, Japan) were produced on a C57BL/6 background. Wild-type (WT) male C57BL/6 mice were purchased from Shanghai Laboratory Animal Center (Shanghai, China). All experiments were performed in accordance with the guidelines established by the Animal Care and Use Committee of Fourth Military Medical University (Xian, China).

### SCI Model

Mice received a severe midthoracic (T8–T9) crush injury using Dumont type forceps with a spacer of 0.2 mm, as described previously [18]. Briefly, laminectomy of vertebrae T8–T9 was performed using a pair of fine rongeurs, and care was taken not to damage the dura. For each mouse, the forceps were used to laterally compress the spinal cord at a depth of 0.2 mm for 20 s. After surgery, each mouse was housed separately, and manual bladder expression was performed twice a day. Sham-operated mice received laminectomy without crush injury.

### Bone Marrow-Derived Macrophages and Drug Treatment

Bone marrow-derived macrophages (BMDMs) were generated, as described previously [19]. Briefly, bone marrow cells from the femurs and tibias of mice were triturated (using 26-gauge needles) in RPMI 1640 medium containing 10 % fetal bovine serum (FBS; Gibco, Carlsbad, CA, USA). Red blood cells were lysed in lysis buffer (0.15 M NH_4_Cl, 10 nM KHCO_3_, and 0.1 mM Na_2_ ethylenediaminetetraacetic acid, pH7.4), washed once in RPMI 1640 with 10 % FBS, and then plated in RPMI 1640 supplemented with 1 % penicillin/streptomycin, 1 % 4-(2-hydroxyethyl) piperazine-1-ethanesulfonic acid, 0.1 % β-mercaptoethanol, 10 % FBS, and 20 % sL929-conditioned medium containing macrophage colony-stimulating factor (M-CSF). After 7–10 days in culture, nonadherent cells were removed and adherent cells harvested for assays to examine the functional capabilities of macrophages under different external stimulation conditions, as described previously [20]. To promote polarization into M1 or M2 macrophages, BMDMs were treated with LPS (100 ng/ml; Sigma-Aldrich, St. Louis, MO, USA) plus IFN-γ (20 ng/ml; Peprotech, Rocky Hill, NJ, USA) or IL-4 (20 ng/ml; Peprotech) for 24 h, respectively. Supernatants from stimulated macrophages were collected for further analysis. The control group received no treatment except a change of medium.

### Primary Microglial Cultures

Microglial cells were isolated from neonatal (1–3-day-old) C57BL/6 mice and PD-1 KO mice, as described previously [21]. Briefly, the cerebral cortex was dissected and the meninges carefully removed under a microscope. The cerebral cortical tissue was then minced and digested with 0.125 % trypsin. Cells were plated onto tissue culture T-75 flasks and in Dulbecco’s modified Eagle medium (DMEM)/F12 (Gibco) containing 10 % FBS and 2 mM L-glutamine. After 14–18 days in culture, microglial cells were collected by shaking the flasks at 220 rpm/min for 3–6 h. Immunostaining for CD11b and ionized calcium-binding adapter molecule 1 (Iba1) confirmed the purity of the culture as >95 % microglial. Microglial cells were cultured in 6-well culture-plates at a density of 1 × 10^6^ cells per well. To promote polarization into the M1 or M2 phenotype, microglia were treated with LPS (100 ng/ml) plus IFN-γ (20 ng/ml) or IL-4 (20 ng/ml), respectively, for 24 h. The control group received no treatment except for a change of medium.

### Quantitative Reverse Transcription Polymerase Chain Reaction

Total RNA was prepared from macrophages/microglia or an 8-mm length of spinal cord tissue from the injured site by lysis in TRIzol (Invitrogen, Carlsbad, CA, USA) following the manufacturer’s instructions. After total RNA was reverse transcribed, the complementary DNA was used to amplify *Pd1*, *Pdl1*, *inos*, *Arg1*, *Cd206*, *Il1b*, *Il4*, *Il10*, *Il12*, *Tnfa*, *Tgfb*, and *Actb*. Reverse transcription polymerase chain reaction (PCR) was performed using the SYBR Premix Ex Taq™IIkit (DRR036A; TaKaRa, Otsu, Japan) and Bio-RadCFX 96 Real-time PCR system (Bio-Rad Laboratories, Hercules, CA, USA), with β-actin used as a reference control. The primers used in the PCRs are shown in Table 1.

**Table 1:** Polymerase chain reaction primer sequences

Gene	Forward primer (5′-3′)	Reverse primer (5′-3′)
*Pd1*	GCCACCTTCACCTGCAGCTTGT	AAACCGGCCTTCTGGTTTGGGC
*Pdl1*	TCACGCTGAAAGTCAATGCCCCA	TCCCACTCACGGGTTGGTGGT
*inos*	CCCTTCAATGGTTGGTACATGG	ACATTGATCTCCGTGACAGCC
*Arg1*	GAACACGGCAGTGGCTTTAAC	TGCTTAGCTCTGTCTGCTTTGC
*Cd206*	TCTTTGCCTTTCCCAGTCTCC	TGACACCCAGCGGAATTTC
*Il1b*	CTTCAGGCAGGCAGTATCAC	CCAGCAGGTTATCATCATCATCC
*Il4*	GGTCTCAACCCCCAGCTAGT	GCCGATGATCTCTCTCAAGTGAT
*Il10*	GCTCTTACTGACTGGCATGAG	CGCAGCTCTAGGAGCATGTG
*Il12*	CTTCTGTCAACACCATCTCT	CTGTGCCTTGGTAGCATCTATG
*Tnfa*	ACACCATGAGCACAGAAAGC	GCCACAAGCAGGAATGAGAAG
*Tgfb*	CTCCCGTGGCTTCTAGTGC	GCCTTAGTTTGGACAGGATCTG
*Actb*	AGAAGGACTCCTATGTGGGTGA	CATGAGCTGGGTCATCTTTTCA

### Western Blotting

Ice-cold lysis buffer (200 μl; 50 mM Tris-HCl, 5 mM ethylenediaminetetraacetic acid, 150 mM NaCl, 0.5 % deoxysodium cholate, 0.1 % sodium dodecyl sulfate, 1 mM dithiothreitol, pH 8.0) containing the protease inhibitors aprotinin (20 μg/ml), phenyl methanesulfonyl fluoride (1 mM), sodium fluoride (5 mM), and sodium orthovanadate (1 mM) was added to each well. The spinal cord tissue samples were collected and then homogenized (10 μl/μg). The homogenates were centrifuged at 15,000 *g* for 10 min at 4 °C and the supernatants collected. Protein concentration was determined using the Bradford method. Samples were boiled for 10 min and proteins separated on 8–15 % sodium dodecyl sulfate polyacrylamide gels via electrophoresis (110 V) and transferred (20 V, overnight) onto polyvinylidene fluoride membranes (Millipore, Billerica, MA, USA), which were then blocked with 5 % nonfat milk for 1 h at room temperature. Membranes were incubated (16–24 h at 4 °C) with the primary antibody for iNOS (1:1000; Abcam, Cambridge, UK), Arg1 (1:1000; Santa Cruz Biotechnology, Santa Cruz, CA, USA), STAT1/p-STAT1 (both 1:1000; Cell Signaling, Danvers, MA, USA), STAT6/p-STAT6 (both 1:1000, Santa Cruz Biotechnology), or nuclear factor-kappa B (NF-κB) (P65)/p-P65 (both 1:1000; Cell Signaling). Membranes were then washed with Tris-buffered saline containing Tween 20 and incubated with the secondary antibody conjugated with horseradish peroxidase (1:8000; Jackson ImmunoResearch, West Rove, PA, USA) for 2 h at room temperature. Bands were visualized with Electro Chemical Luminescence (ECL) solution and the Bio-Rad Image Lab system. The densities of specific bands were measured with Image J software (NIH, Bethesda, MD, USA).

### Enzyme-Linked Immunosorbent Assay

Macrophage/microglial culture supernatants were collected after treatment to measure the secreted inflammatory mediators using IL-12, IL-10, TNF-α, and IFN-γ enzyme-linked immunosorbent assay (ELISA) kits (R&D Systems, Minneapolis, MN, USA), according to their respective protocols.

### Flow Cytometry

Microglial cells (1 × 10^6^) were first incubated in rat serum and antimouse CD16/CD32 (1:50; BD Pharmingen, San Jose, CA, USA). Fluorescein isothiocyanate (FITC)-conjugated rat antimouse PD-1 (1:100; eBioscience, San Diego, CA, USA) and phycoerythrin (PE)-conjugated rat antimouse PD-L1 (1:100; eBioscience) or isotype-matched antibodies (Sigma-Aldrich) were then added for 30 min at 4 °C. Cells were washed with fluorescence-activated cell sorting solution [phosphate-buffered saline (PBS) containing 1 % fetal bovine serum and 0.05 % NaN_3_] and then resuspended in 200 μl fluorescence-activated cell sorting solution. For cytoplasmic staining, 4 μl BD golgistop solution (BD Pharmingen) was added for every 6 ml of cell culture and thoroughly mixed during the last 6 h of stimulation. Intracellular cytokines were assayed following the recommended protocols (BD Cytofix/Cytoperm Plus Fixation/Permeabilization Kit; BD Pharmingen). FITC-conjugated antimouse IFN-γ (1:100; eBioscience) and PE-conjugated antimouse Il-4 (1:100; eBioscience) were used for this assay. Flow cytometry analysis was performed using the Millipore flow cytometer (Guawa 6HT). The data were analyzed using FlowJo software v. 7.6.2 (TreeStar, Ashland, OR, USA).

### Phagocytosis Assay

Carboxylate-modified red fluorescent latex beads (2 μm diameter; 4 μl of 2.5 % latex bead solution; Sigma-Aldrich) were opsonized in 10 % FBS with DMEM or DMEM/DF12 (6 ml) for 60 min at 37 °C. Subsequently, cells were washed twice with ice-cold PBS to remove uningested particles, and fixed with 4 % paraformaldehyde. The fluorescence of macrophages or microglia were measured by flow cytometry. Results were presented as a percentage of the phagocytic cell population (number of cells that ingest at least 1 bead/total number of cells × 100 %) and phagocytosis index (average number of ingested beads per cell = number of ingested beads/total number of cells).

### Immunofluorescence

BMDMs and microglial cells were fixed with 4 % paraformaldehyde in 0.1 M phosphate buffer (pH 7.4) for 15 min, then blocked for 1 h with 1 % bovine serum albumin containing 0.3 % Triton X-100, followed by incubation (overnight at 4 °C) with the following primary antibodies: goat antimouse PD-L1 (1:100; R&D Systems), rat antimouse PD-1 (1:100; AbD Serotec, Oxford, UK), rabbit antimouse Iba1 (1:1000; Wako, Osaka, Japan), After cells were washed with PBS, they were incubated (for 2 h at room temperature) with their respective secondary antibody: Dylight (Dy)488- and Dy594-conjugated secondary antibodies (all 1:1000; Jackson ImmunoResearch, West Grove, PA). Images were acquired using FV 1000 confocal microscope (Olympus, Tokyo, Japan).

### Immunohistochemistry

Fourteen days after SCI, animals were perfused through the left cardioventricle with 10 ml of physiological saline followed by 40 ml of 4 % paraformaldehyde in 0.1 M phosphate buffer. Spinal cord tissues were postfixed for 4–6 h and immersed in 25 % sucrose solution for 24–48 h at 4 °C until they sank. Sections were cut (12 μm thickness) with a cryostat and thaw-mounted onto gelatinized slides followed by blocking with 1 % bovine serum albumin containing 0.3 % Triton X-100 for 1 h. Sections were then incubated (overnight at 4 °C) with the following primary antibodies: goat antimouse PD-L1 (1:100; R&D Systems), rat antimouse PD-1 (1:100; AbD Serotec), rabbit antimouse Iba1 (1:1000; Wako), goat antimouse Arg1 (1:100; Santa Cruz Biotechnology), rabbit antimouse iNOS (1:200; Abcam), rat antimouse F4/80 (1:100; AbD Serotec). The next steps for single, double, and triple staining were performed using standard immunocytochemical methods. The sections were then incubated (for 2 h at room temperature) with the respective secondary antibodies and the following direct fluorescent antibodies: FITC-conjugated rat antimouse PD-1 (1:100; eBioscience), PE-conjugated rat antimouse PD-L1 (1:100; eBioscience), allophycocyanin-conjugated rat antimouse F4/80 (1:100; eBioscience). Dy488-, Dy594-, and Dy649-conjugated secondary antibodies (all 1:1000; Jackson ImmunoResearch) were used to visualize the primary antibodies. Images were acquired with a FV 1000 confocal microscope (Olympus).

### Open Field Locomotion

The Basso Mouse Scale (BMS) score was used to assess locomotor behavior of mice after SCI. The animals were placed in an open field (80 × 130 × 30 cm) free of any tactile stimulation, and the test was performed according the rules of the BMS score [22]. To assess their performance using the BMS score, each animal was observed by two evaluators (blinded to the treatment) for 4 min.

### Statistical Analysis

All data are presented as the mean ± standard error of the mean (unless otherwise stated). Independent samples *t* tests were used to compare the two groups separately. Analysis of variance followed by the Tukey–Kramer test was used for multiple comparisons. Statistical analysis was performed with the OriginPro 7.0 program. Significance was reached at values of *p* < 0.05 or *p* < 0.001.

## Results

### PD-1 is Upregulated in Macrophages/Microglia After SCI, and PD-1 Deficiency Causes Macrophages/Microglia to Polarize to M1-type Cells in Injured Spinal Cords in Mice

**Fig. 1:**
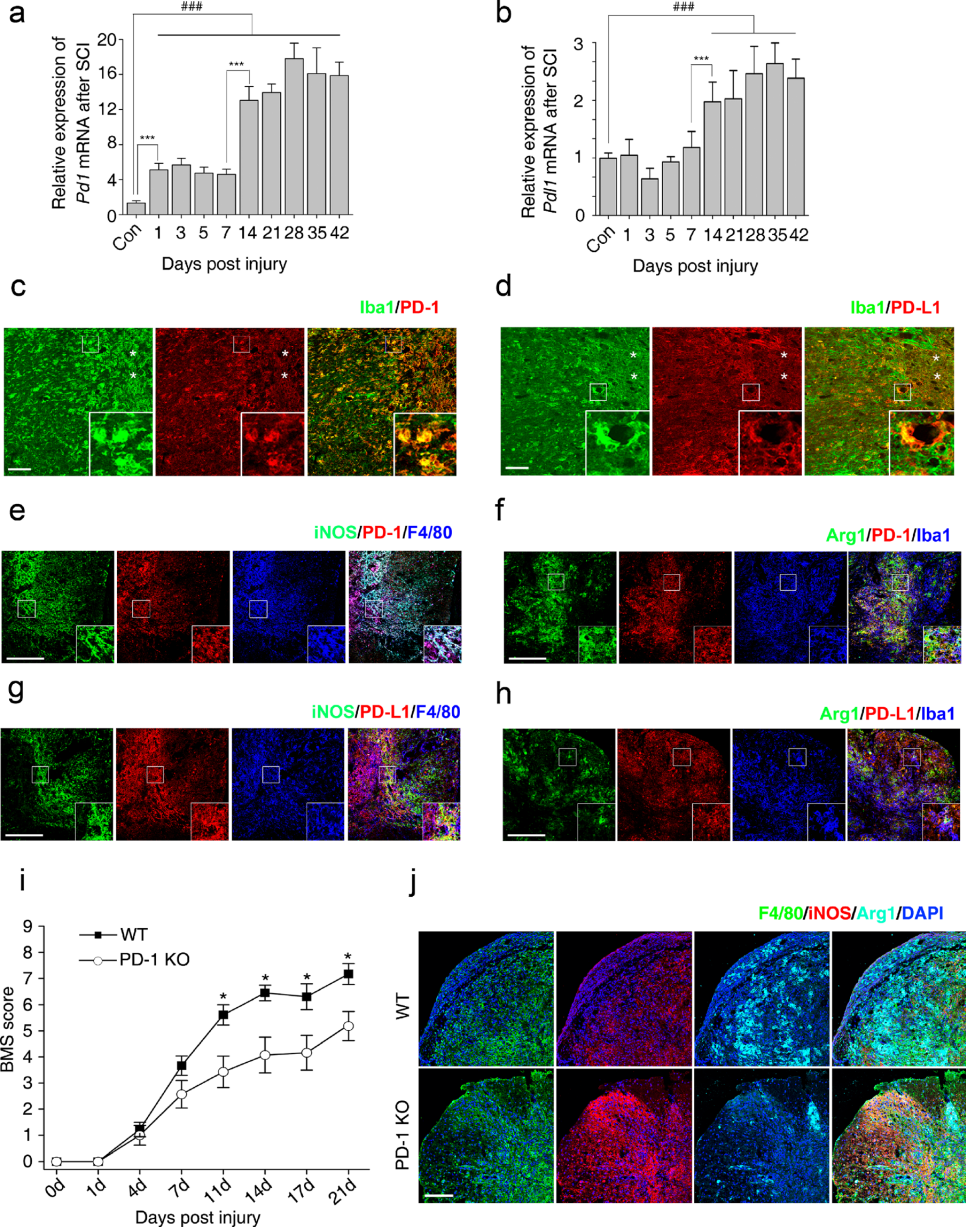
Expression of programmed death-1 (PD-1) in macrophages/microglia after spinal cord injury (SCI) in mice. Quantitative reverse transcription polymerase chain reaction at each timepoint after SCI for (a) *Pd1* (*n* = 3 mice in each group) and (b) *Pdl1* (*n* = 3 mice in each group). Immunohistochemistry (IHC) in macrophages/microglia 14 days postinjury (dpi) in wild-type (WT) mice for (c) PD-1, (d) it’s ligand, PD-L1, and (e) PD-1 in M1 cells [inducible nitric oxide synthase (iNOS)^+^]; (f) PD-1 in M2 cells [arginase 1 (Arg1^+^)]; (g) PD-L1 in M1 cells (iNOS^+^); and (h) PD-L1 in M2 cells (Arg1^+^). The injury epicenter in (c) and (d) are denoted with an asterisk. (i) Locomotor recovery evaluated by the Basso Mouse Scale (BMS) score acquired at different timepoints after SCI in WT and PD-1^–/–^ mice (*n* = 8 mice). (j) IHC for iNOS and Arg1 in macrophages/microglia at 14 dpi. (a, b) ^###^ *p* < 0.001 *versus* control, ***p < 0.001 *versus* the previous timepoint. (i) *p < 0.05 *versus* WT mice. Scale bar = (c–h) 100 μm and (j) 200 μm. KO = knockout; Iba1 = ionized calcium-binding adapter molecule-1; DAPI = 4’,6-diamidino-2-phenylindole

Most studies on the role of PD-1 in the central nervous system (CNS) have focused on experimental autoimmune encephalomyelitis (EAE), which is a prototypical autoimmune disease of the CNS [23–25]. However, there are almost no reports on the function of PD-1 in SCI that include macrophages/microglia as the major inflammatory cells that determine functional recovery [1, 2]. We first performed quantitative reverse transcription PCR to quantify the expression of *Pd1* mRNA at different time points after SCI. *Pd1* mRNA increased in 2 phases 42 days after SCI (Fig. 1a). *Pd1* mRNA increased 5-fold (5.11 ± 0.75) compared with control mice 7 days after SCI. *Pd1* mRNA in macrophages increased more than 13-fold (13.07 ± 1.58) from 14 and 42 days after injury (Fig. 1a). The highest fold increase (17.80 ± 1.78) was observed 28 days after SCI. We also quantified the mRNA levels of *Pdl1. Pdl1* mRNA increased approximately 1-fold 14 days after injury (Fig. 1b). Immunohistochemistry (IHC) revealed enhanced expression of both PD-1 (Fig. 1c) and PD-L1 (Fig. 1d) in macrophages/microglia localized in the lesion areas after SCI. However, neither PD-1 nor PD-L1 was detected in normal spinal cord sections (data not shown). Furthermore, the presence of PD-L1, but not PD-1, was observed in astrocytes around the lesion sites after SCI (Supplementary Fig. S1). These results suggest that both PD-1 and PD-L1 are upregulated after SCI.

To detect whether PD-1 and PD-L1 molecules are also expressed in polarized macrophages/microglia, triple IHC was used to detect PD-1 and PD-L1 in M1 (iNOS^+^; Fig 1e, g) or M2 (Arg1^+^) cells (Fig. 1f, h). PD-1 and PD-L1 were found to be present in M1 and M2 cells in injured spinal cord at 14 days postinjury (dpi) in WT mice (Fig. 1e–h).

Next, we used a severe crush model of SCI to determine locomotor recovery in both PD-1-KO and WT mice. Although PD-1-KO mice had low BMS scores 1 week after injury, they were not significantly different compared with WT mice (Fig. 1i). In contrast, PD-1-KO mice showed poor locomotor recovery 2 and 3 weeks after SCI compared with WT mice, indicating that PD-1 deficiency delays locomotor recovery after SCI.

Polarized macrophages/microglia determine locomotor recovery after SCI [3, 7]. To assess whether PD-1 deficiency induces the polarization of macrophages and microglia *in vivo*, we used antibodies against specific M1 and M2 markers to detect these cells in spinal cord sections by IHC after SCI in both WT and PD-1-KO mice. At 14 dpi, the number of iNOS-positive macrophages/microglia (M1 cells) was higher in PD-1 KO than in WT mice, whereas the number of Arg1-positive macrophages/microglia (M2 cells) was lower in PD-1 KO mice than in WT mice (Fig. 1j). These results suggest that, after SCI, PD-1 deficiency causes macrophages/microglia to polarize to M1-type cells.

### PD-1 is Upregulated in Cultured Macrophages and Microglia Under Polarized Stimulation

**Fig. 2:**
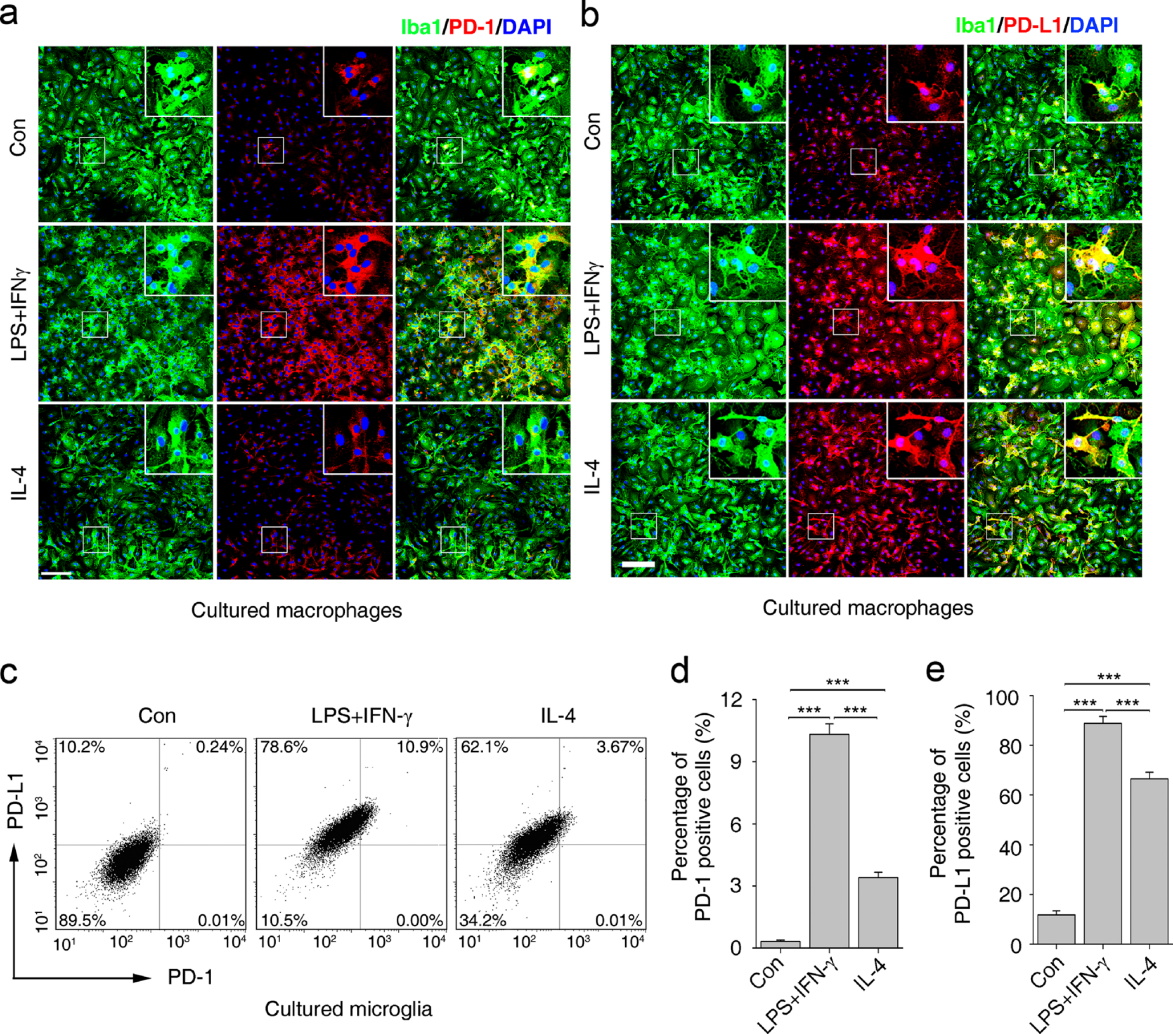
Quantification of programmed death-1 (PD-1) and it’s ligand, PD-L1, in cultured macrophages and microglia under polarized stimulation. The presence of PD-1 (a) and PD-L1 (b) in cultured macrophages respectively, under lipopolysaccharide (LPS) + interferon-gamma (IFN-γ) or interleukin (IL)-4 stimulation. (c) Flow cytometry of PD-1 and PD-L1 in cultured microglia under LPS + IFN-γ or IL-4 stimulation. Percentage of (d) PD-1- and (e) PD-L1-positive cells in polarized microglia. Scale bar = 100 μm. *n* = 3 mice in each group. ***p < 0.001. Con = control; Iba1 = ionized calcium-binding adapter molecule-1; DAPI = 4’,6-diamidino-2-phenylindole

To evaluate whether expression of PD-1 and PD-L1 is regulated by inflammatory cytokines in macrophages and microglia, we stimulated cultured BMDMs and primary microglia with LPS + IFN-γ or IL-4, which are the classical stimuli used to polarize macrophages into the M1 or M2 phenotype, respectively [4]. The level of PD-1 was higher in BMDMs stimulated with LPS + IFN-γ compared with those stimulated with IL-4 (Fig. 2a). PD-1 was weakly present in control BMDMs (Fig. 2a). PD-L1 was also upregulated in BMDMs under LPS + IFN-γ or IL-4 stimulation. Similarly to PD-1, the presence of PD-L1 was higher in BMDMs stimulated with LPS + IFN-γ (Fig. 2b). These results suggest that the expression levels of PD-1 and PD-L1 are increased in both M1 and M2 macrophages.

The results from IHC (data not shown) and flow cytometry analysis revealed an increased expression of both PD-1 and PD-L1 in primary microglia stimulated with LPS + IFN-γ or IL-4 (Fig. 2c–e). PD-1 expression was induced in approximately 10.3 ± 0.51 % and 3.4 ± 0.25 % of microglial cells with a 24-h treatment of LPS + IFN-γ and IL-4 (Fig. 2c, d), respectively. The expression of PD-L1 was higher than that of PD-1. In the LPS + IFN-γ group, 88.8 ± 2.80 % of microglia expressed PD-L1 compared with 66.5 ± 2.70 % in the IL-4 group (Fig. 2c, e). These results show that the expression PD-1 and PD-L1 is induced in macrophages and microglia under polarized stimulation, suggesting that PD-1 may play a role in the regulation of the polarization of macrophages and microglia.

### PD-1 Deficiency Improved M1 Polarization of Cultured Macrophages

**Fig. 3:**
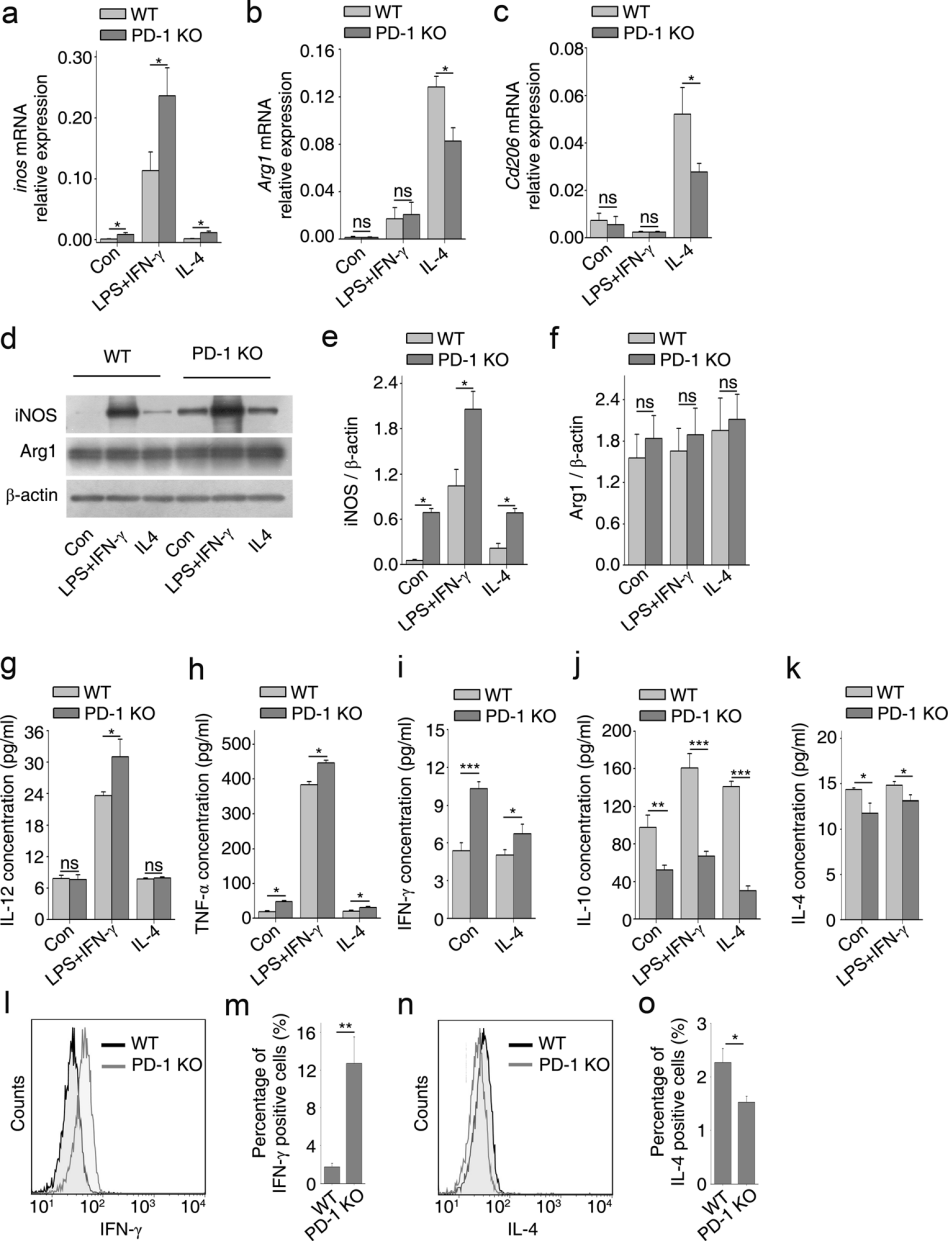
Deficiency of programmed death-1 (PD-1) deficiency improves M1 polarization of cultured macrophages. Bone marrow-derived macrophages (BMDMs) from wild-type (WT) and PD-1-knockout (KO) mice were stimulated with lipopolysaccharide (LPS) + interferon-gamma (IFN-γ) or interleukin (IL)-4 for 24 h followed by quantitative reverse transcription polymerase chain reaction for (a) *inos*, (b) *Arg1*, and (c) *Cd206*, and Western blotting (d) for inducible nitric oxide synthase (iNOS) and arginase 1 (Arg1). Densitometric analysis of protein bands for (e) iNOS and (f) Arg1 normalized to β-actin. Enzyme-linked immunosorbent assays for the proinflammatory cytokines: (g) IL-12, (h) tumor necrosis factor-alpha (TNF-α), and (i) IFN-γ, and the anti-inflammatory cytokines: (j) IL-10 and (k) IL-4. Cytoplasmic (l) IFN-γ and (n) IL-4 in BMDMs measured by flow cytometry for (m) IFN-γ and (o) IL-4, respectively. *n* = 3 mice in each group. *p < 0.05, **p < 0.01, ***p < 0.001. Con = control

iNOS and Arg1/mannose receptor (CD206) were used as characteristic markers for M1 and M2 polarization, respectively [7, 26]. In BMDMs from PD-1-KO mice and those stimulated with LPS + IFN-γ or IL-4, the levels of *inos* mRNA were significantly increased compared with control BMDMs (Fig. 3a). However, the mRNA levels of *Arg1* and *Cd206* were significantly reduced in IL-4-stimulated BMDMs (Fig. 3b, c). Protein levels of iNOS in PD-1-deficient BMDMs were also upregulated in polarized stimulations (Fig. 3d, e), while Arg1 in cytokine-stimulated BMDMs did not differ from those of control (Fig. 3d, f), which may have been caused by the treatment of these cells with M-CSF during BMDM differentiation. M-CSF can induce the expression of part of the M2 transcriptome, suggesting that a default shift towards the M2 phenotype may occur under homeostatic conditions [6].

We also measured the levels of the proinflammatory cytokines (M1 polarization) IL-12 (Fig. 3g), TNF-α (Fig. 3h), and IFN-γ (Fig. 3i), and the anti-inflammatory cytokines (M2 polarization) IL-10 (Fig. 3j) and IL-4 (Fig. 3k) by ELISA and flow cytometry. ELISA assays showed that macrophages from PD-1 KO mice produced higher levels of proinflammatory cytokines than WT mice (Fig. 3g–i); however, the levels of anti-inflammatory cytokines were decreased (Fig. 3j, k). The levels of IFN-γ and IL-4 in the IFN-γ + LPS- and IL-4-treated groups were not measured because both cytokines were added to the culture medium to polarize BMDMs. Instead, we quantified the amount of cytoplasmic IFN-γ and IL-4 in these groups via immunostaining with the corresponding antibodies. We then analyzed them by flow cytometry (Fig. 3l–o). As expected, macrophages from PD-1 KO mice expressed more IFN-γ in the LPS + IFN-γ -treated group (Fig. 3l, m); however, IL-4 was reduced in the IL-4 group (Fig. 3n, o). These results suggest that PD-1 deficiency shifts the macrophage population towards M1 polarization instead of M2 polarization.

### PD-1 Deficiency Improves the M1 Polarization of Cultured Microglia

**Fig. 4:**
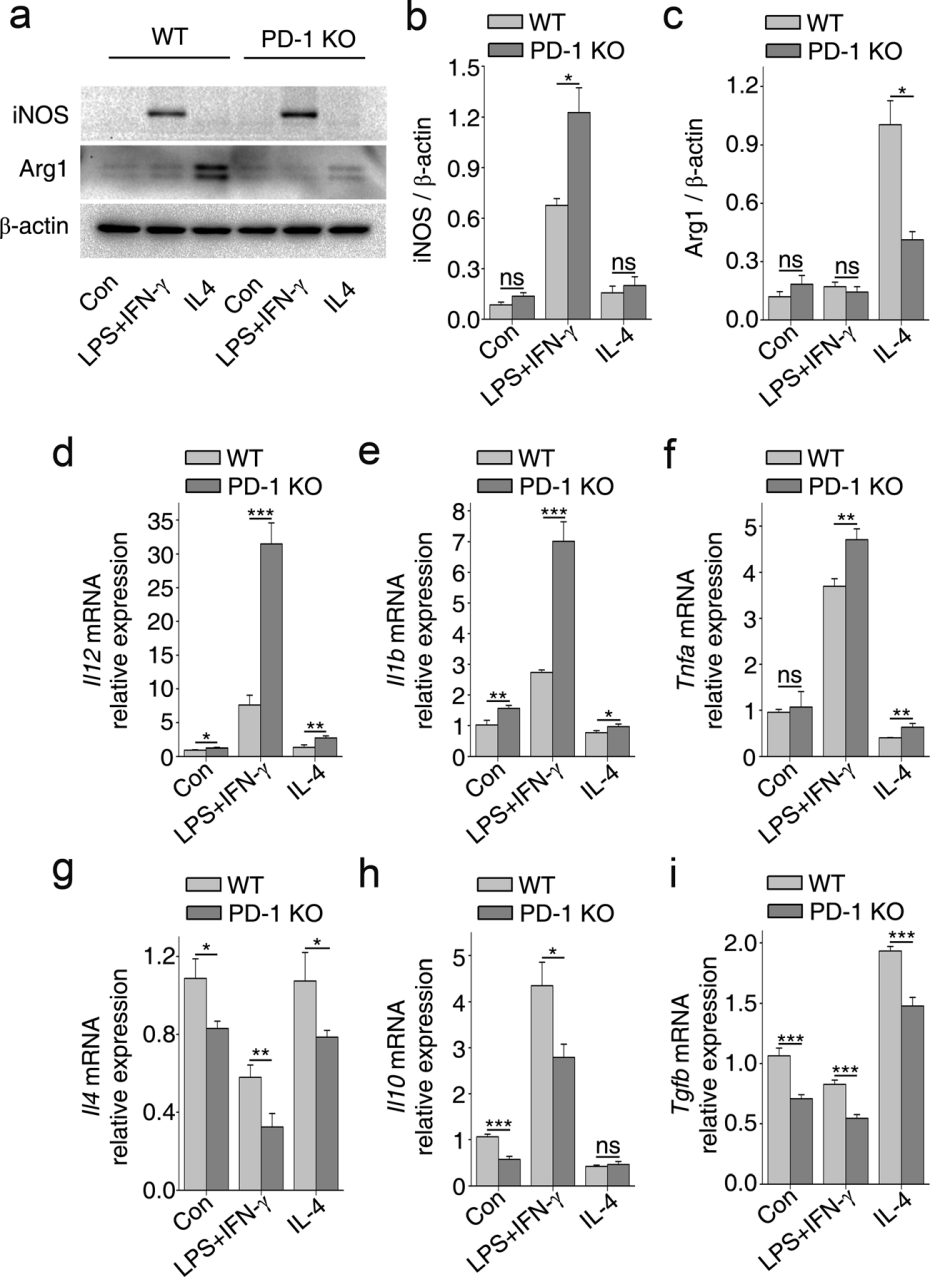
Deficiency of programmed death-1 (PD-1) deficiency enhances M1 polarization of cultured microglia. (a) Western immunoblot for inducible nitric oxide synthase (iNOS; M1 phenotype) and arginase 1 (Arg1) (M2 phenotype) in primary microglial cells isolated from wild-type (WT) and PD-1-knockout (KO) mice and stimulated with lipopolysaccharide (LPS) + interferon (IFN)-γ or interleukin (IL)-4 for 24 h. Densitometric analysis of (b) iNOS and (c) Arg1 normalized to β-actin. Quantitative reverse transcription polymerase chain reaction of proinflammatory cytokines (d) *Il12*, (e) *Il1b*, and (f) *Tnfa*; anti-inflammatory cytokines (g) *Il4*, (h) *Il10*, and (i) *Tgfb. n* = 3 mice in each group. *p < 0.05, **p < 0.01, ***p < 0.001. Con = control; ns = not significant

We also examined the effects of PD-1 on the polarization of primary microglia isolated from PD-1 KO and WT mice. Microglial cells from PD-1-KO mice exhibited an enhanced M1 response when treated with LPS + IFN-γ, as shown by an increase in the protein expression of iNOS compared with controls (Fig. 4a, b). In contrast, Arg1 was reduced in the IL-4 group, indicating an attenuated M2 response (Fig. 4a, c). Analysis of mRNA expression showed that in microglia from PD-1-KO mice, the expression levels of *Il12* (Fig. 4d), *Il1b* (Fig. 4e), *and Tnfa* (Fig. 4f) under LPS + IFN-γ stimulation were higher than in WT mice (Fig. 4d–f); however, the expression levels of *Il4* (Fig. 4g), *Il10* (Fig. 4h), *and Tgfb* (Fig. 4i) were suppressed. These results indicate that, in a similar manner to macrophages, PD-1 deficiency shifts microglial polarization towards the M1 phenotype instead of the M2 phenotype.

### PD-1 Deficiency Influences the Polarization of Cultured Macrophages/Microglia via STAT1 and STAT6

**Fig. 5:**
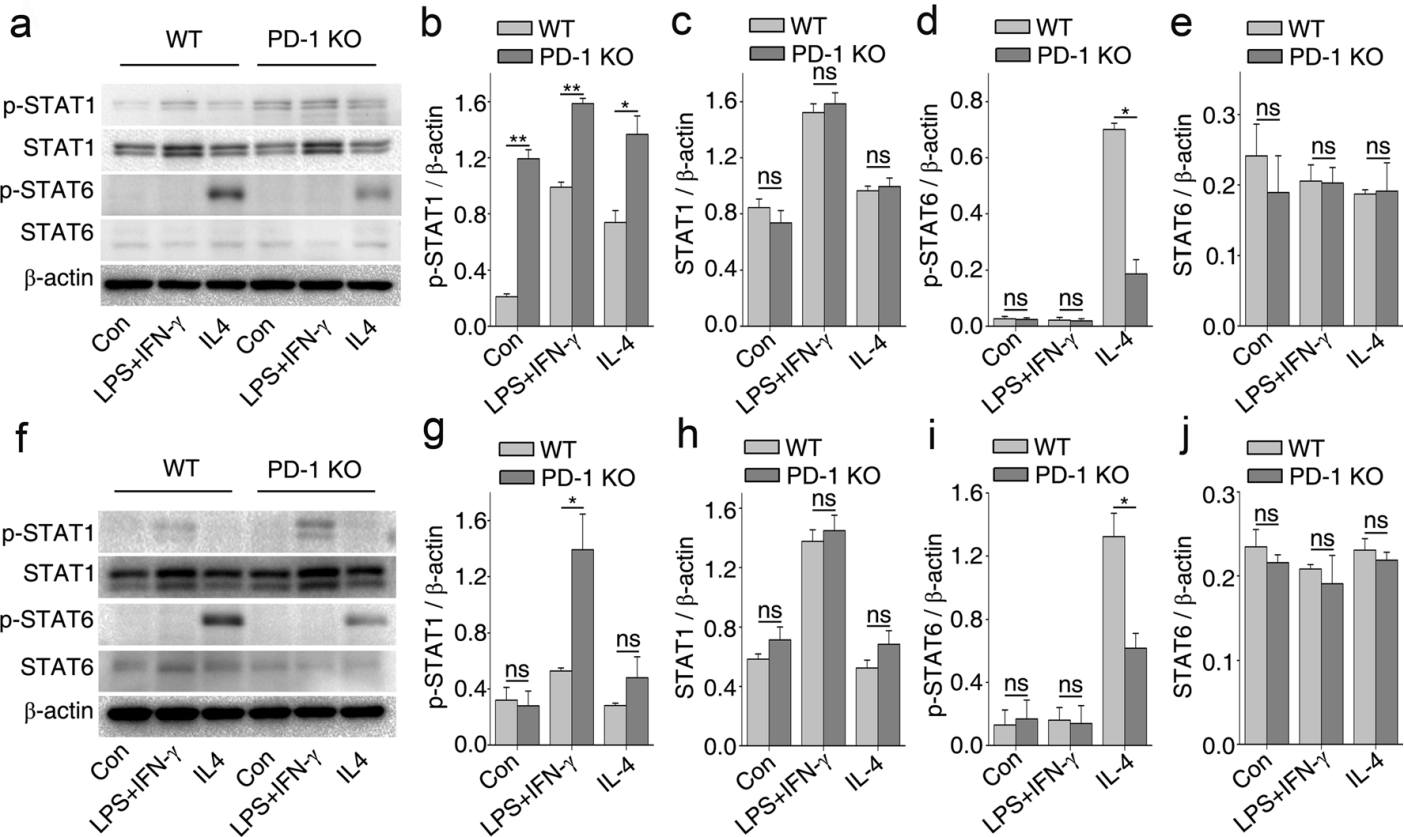
Deficiency in programmed death-1 (PD-1) deficiency influences the polarization of macrophages and microglia via signal transducer and activator of transcription (STAT)1 and STAT6. (a) Western immunoblot for phosphorylated (p)-STAT1, STAT1, p-STAT6, and STAT6 in macrophages stimulated with lipopolysaccharide (LPS) + interferon (IFN)-γ or interleukin (IL)-4 for 24 h. Band intensities of (b) p-STAT1, (c) STAT1, (d) p-STAT6, and (e) STAT6 normalized to β-actin. (f) Western immunoblot for p-STAT1, STAT1, p-STAT6, and STAT6 in microglial cells stimulated with LPS + IFN-γ or IL-4 for 24 h. Densitometric analysis for (g) p-STAT1, (h) STAT1, (i) p-STAT6, and (j) STAT6 normalized to β-actin. *n* = 3 mice in each group. *p < 0.05, **p < 0.01. Con = control; ns = not significant

IFN-γ promotes the M1 phenotype in macrophages mainly through the Janus kinase (JAK)-STAT1 signaling pathway, whereas IL-4 promotes the M2 phenotype in macrophages mainly through the JAK-STAT6 signaling pathway [3, 27]. Because STAT1 and STAT6 have a reciprocal inhibitory relationship in macrophage polarization [3, 27], our next aim was to determine if PD-1 regulates M1 and M2 polarization through the STAT1 and STAT6 pathways, respectively. Levels of phosphorylated (p)-STAT1 were significantly increased in BMDMs from PD-1-KO with or without polarized stimulation compared with the levels in WT BMDMs (Fig. 5a, b). In contrast, the level of p-STAT6 was significantly decreased in PD-1-KO BMDMs under IL-4 stimulation as compared with WT BMDMs (Fig. 5a, d). In microglia isolated from PD-1-KO mice, an enhanced level of p-STAT1 was found only in the LPS + IFN-γ-treated group (Fig. 5f, g) compared with WT microglia. No change in the level of p-STAT1 was observed in PD-1-KO microglia stimulated with IL-4 (Fig. 5f, g). However, p-STAT6 was significantly decreased in this group (Fig. 5f, i). Our results indicate that PD-1 deficiency promotes M1 rather than M2 polarization of macrophages/microglia by enhancing the expression of p-STAT1 and downregulating p-STAT6.

### PD-1 Deficiency Influences the Polarization of Macrophages/Microglia via STAT1 and NF-κB Under *In Vivo* Conditions

**Fig. 6:**
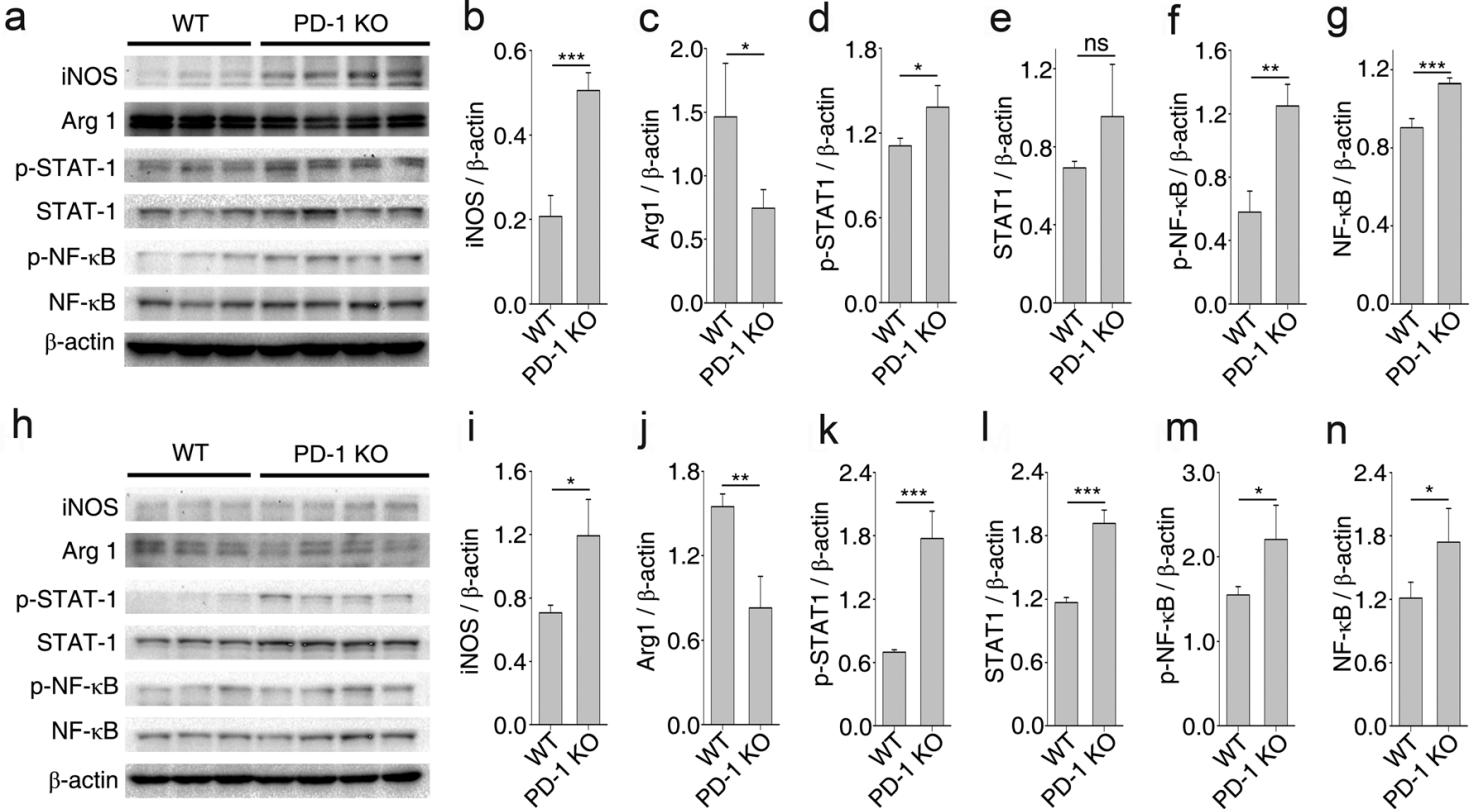
Mechanism of programmed death-1 (PD-1) deficiency influences the polarization of macrophages/microglia *in vivo* after spinal cord injury. (a) Western immunoblot of inducible nitric oxide synthase (iNOS), arginase 1 (Arg1), phosphorylated signal transducer and activator of transcription (p-STAT1), signal transducer and activator of transcription (STAT1), phosphorylated nuclear factor kappa-B (p-NF-κB), and nuclear factor kappa-B (NF-κB) in wild-type (WT) (*n* = 3) and PD-1-knockout (KO) mice (*n* = 4) 14 days postinjury (dpi). Densitometric analysis for (b) iNOS, (c) Arg1, (d) p-STAT1, (e) STAT1, (f) p-NF-κB, and (g) NF-κB relative to β-actin. (h) Western immunoblot of iNOS, Arg1, p-STAT1, STAT1, p-NF-κB, and NF-κB in WT mice (*n* = 3) and PD-1-KO mice (*n* = 4) 21 dpi. Densitometric analysis for (i) iNOS, (j) Arg1, (k) p-STAT1, (l) STAT1, (m) p-NF-κB, and (n) NF-κB relative to β-actin. *p < 0.05, **p < 0.01, ***p < 0.001. ns = not significant

To further confirm that a deficiency in PD-1 induces the polarization of macrophages/microglia, we quantified the protein expression levels of iNOS and Arg1 in samples extracted from injured spinal cord tissues at 14 and 21 dpi. Consistent with the IHC results shown in Fig. 1, the level of iNOS was significantly higher and Arg1 was lower in PD-1-KO mice compared with the control group. This difference was observed at 14 dpi (Fig. 6a–c). STAT1 and NF-κB are two important transcription factors that regulate the expression of genes characteristic of the M1 phenotype [3, 27]. PD-1 deficiency enhanced the phosphorylation of STAT1 (Fig. 6a, d) and NF-κB (Fig. 6a, f) at 14 dpi in the lesion site, indicating that PD-1 regulates macrophage/microglial plasticity and polarization via different signaling pathways. Similar results were obtained in samples extracted from injured spinal cord tissues at 21 dpi (Fig. 6h–n).

### A Deficiency in PD-1 has Divergent Roles in Macrophages and Microglia in Terms of Phagocytosis

**Fig. 7:**
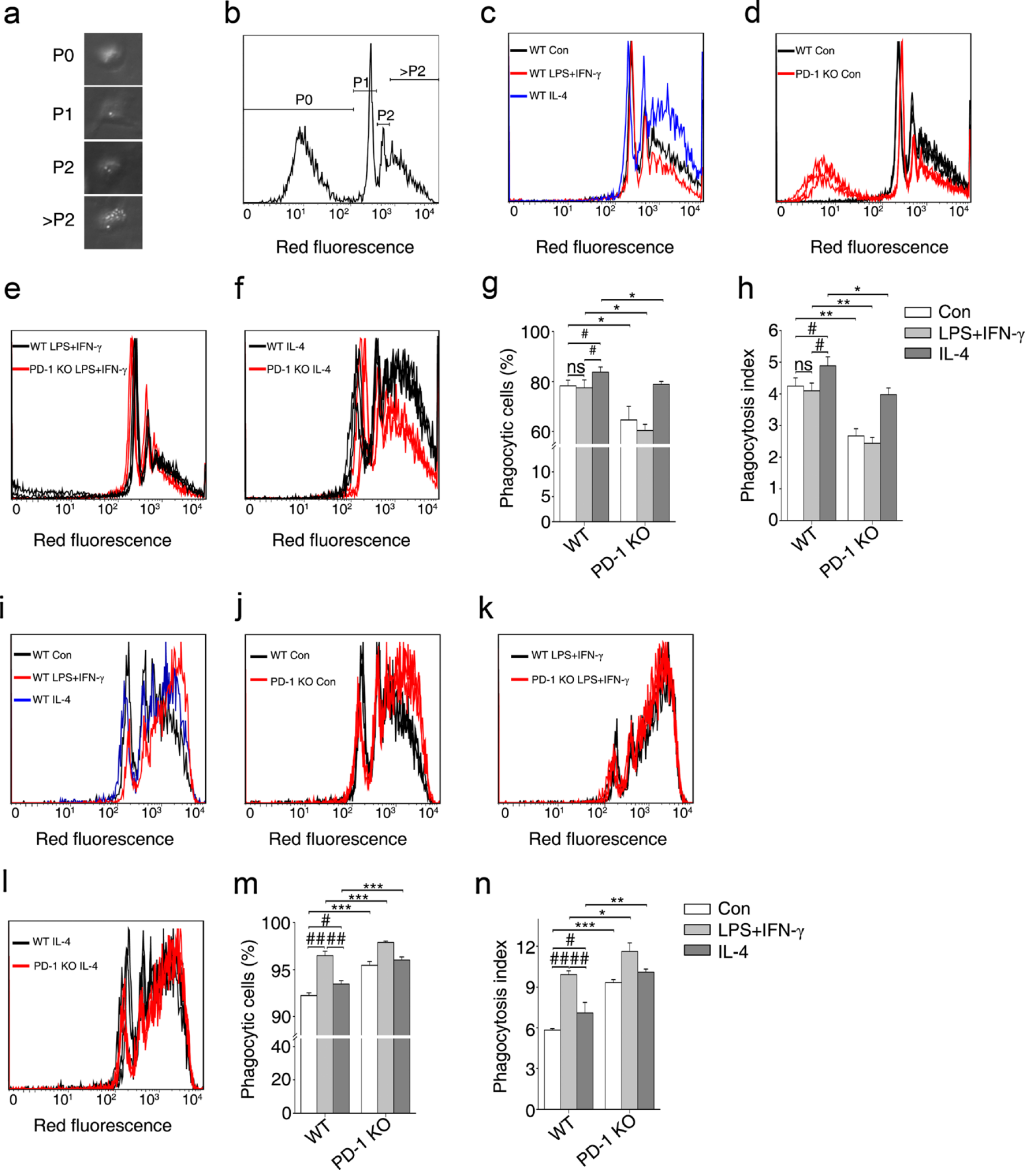
A deficiency in programmed death-1 (PD-1) deficiency promotes opposing phagocytic effects in macrophages and microglia. (a) Representative image under light microscopy showing the morphology of macrophages that did not phagocytize any beads (P0), and phagocytized 1 bead (P1), 2 beads (P2), or numerous beads (>P2). (b) Representative diagram showing macrophages with phagocytized beads by flow cytometric analysis. (c) Phagocytosis of wild-type (WT) macrophages under polarized stimulation analyzed by flow cytometry. Phagocytosis of WT and PD-1-knockout (KO) macrophages with (d) no stimulus, (e) lipopolysaccharide (LPS) + interferon-gamma (IFN-γ), or (f) interleukin (IL)-4. (g) The percentage of phagocytic cells calculated in WT and PD-1-KO macrophages. (h) The phagocytosis index calculated in WT and PD-1-KO macrophages. (i) Phagocytosis of WT microglia under polarized stimulation analyzed by flow cytometry. Phagocytosis of WT and PD-1-KO microglia with (j) no stimulus, (k) LPS + IFN-γ, or (l) IL-4. (m) The percentage of phagocytic cells in WT and PD-1-KO microglia. (n) The phagocytosis index calculated for WT and PD-1-KO microglia. *n* = 3 mice in each group. ^#^ *p* < 0.05, ^##^ *p* < 0.01 *versus* WT, *p < 0.05, **p < 0.01 *versus* WT and PD-1-KO. Con = control

Phagocytosis is the key mechanism of innate immunity, involving the internalization of diverse particulate targets by macrophages, dendritic cells, and other myeloid phagocytes [28]. Macrophages contribute to the balance between antigen availability and clearance through phagocytosis and subsequent degradation [6]. To investigate the role of PD-1 in the regulation of macrophage/microglial phagocytosis under different polarized phenotypes, carboxylate-modified red fluorescent latex beads were incubated with either polarized macrophages or microglia and flow cytometry was then performed to estimate the number of latex beads ingested by each cell (Fig. 7a, b). The percentage of phagocytic cells and the phagocytosis index showed that BMDMs stimulated with IL-4 from the WT group phagocytized more latex beads than those cells from the control group and the LPS + IFN-γ group (Fig. 7c, g, h). Compared with WT BMDMs, PD-1-KO decreased the phagocytic ability of BMDMs with or without polarized stimulation (Fig. 7d–h), while PD-1-KO BMDMs stimulated with IL-4 increased phagocytic ability compared with the control and the LPS + IFN-γ groups (Fig. 7g, h). These results suggest that in WT BMDMs, M2 macrophages possess a better phagocytic ability than M1 macrophages, and that a PD-1 deficiency decreased the phagocytic ability of BMDMs in both the M1 and M2 phenotypes.

Next we studied the effect of PD-1 deficiency on microglial phagocytosis. Interestingly, the characteristics of phagocytosis in microglia were quite different from those in BMDMs. In contrast to BMDMs, LPS + IFN-γ treatment of WT microglia resulted in the highest phagocytic ability compared with the control and IL-4 stimulation groups (Fig. 7i, m, n). However, regardless of the cytokine stimulus used, treatment of PD-1 KO microglia resulted in a better phagocytic ability compared with WT microglia (Fig. 7j–n). These results suggest that in WT-derived microglia, M1-polarized microglial cells possess a better phagocytic ability than M2 cells, and that a PD-1 deficiency enhances phagocytosis by microglia in both the M1 and M2 phenotypes.

In summary, both WT macrophages and microglia were polarized to the M1 or M2 phenotype under LPS + IFN-γ or IL-4 stimulation, respectively. However, their phagocytic abilities were quite different. M2 macrophages had a better phagocytic ability than M1 macrophages, whereas M1 microglial cells had a better phagocytic ability than M2 microglia. In general, a deficiency in PD-1 decreased the phagocytic ability of macrophages and increased microglial phagocytosis, but phagocytic tendencies between WT polarized macrophages and microglia did not change.

## Discussion

PD-1 is a critical co-inhibitory receptor; its gene was first reported to be upregulated in a T-cell hybridoma undergoing cell death [29]. PD-1 and its ligands, PD-L1 and PD-L2, deliver inhibitory signals that regulate the balance between T-cell activation, tolerance, and immunopathology [11]. Most studies on the role of PD-1 in the CNS are focused on EAE [23–25]. A PD-1 deficiency increases the severity of EAE owing to the loss of its inhibitory effect on autoreactive T lymphocytes [23, 24]. Recently, several studies have shown the inducible expression of PD-1 in monocytes/macrophages and dendritic cells [13, 15, 30–32]. However, reports on PD-1 in macrophages/microglia to determine a functional recovery from SCI are scarce [1, 2]. The results of this study show that *PD1* is upregulated following SCI and the PD-1 protein is expressed mainly on macrophages/microglia. However, some PD-1-positive cells negative for Iba-1 were found at the lesion site of the injured spinal cord, suggesting that these cells, which were not identified as neuronal cells (data not shown), are other blood-derived cells, such as lymphocytes [1, 8]. PD-L1 is the most important ligand of PD-1 and is widely expressed in neurons, astrocytes, microglia, and endothelial cells in CNS inflammatory diseases and models such as EAE and multiple sclerosis [24, 33]. Our findings show a high level of expression of PD-L1 in macrophages/microglia and astrocytes at the lesion site of the spinal cord. In addition to its role in the CNS, PD-1 in macrophages has been found to regulate negatively cytokine secretion, cell differentiation, and microbial clearance [13, 15, 16, 30, 32]. Therefore, PD-1 may provide a negative feedback signal to regulate precisely activation, cytokine secretion, and differentiation of macrophages/microglia after SCI.

Macrophages respond to environmental cues (e.g., microbial products, damaged cells, activated lymphocytes), resulting in two opposing functional phenotypes, defined as M1 (the classical proinflammatory macrophage) and M2 (the alternatively activated anti-inflammatory macrophage) [3, 27, 34]. The findings of this study show high expression of both PD-1 and PD-L1 in the treatment of cultured BMDMs and microglial cells with LPS + IFN-γ (for M1 stimulation) and IL-4 (for M2 stimulation), respectively. However, expression of PD-1 was observed in control BMDMs. These cells were cultured with M-CSF, which has been shown to induce the expression of a part of the M2 transcriptome, suggesting that a default shift towards the M2 phenotype may occur under homeostatic conditions [6, 26].

SCI elicits a robust and persistent inflammatory response [1]. Proinflammatory cytokines, reactive oxygen species, metalloproteinases, and danger-associated molecular patterns (such as high-mobility group box 1 protein, histones, and adenosine triphosphate), are released during the early stage of SCI [1, 35, 36]. Under this inflammatory environment, most macrophages/microglia exhibit the M1 phenotype, and the M2 phenotype is displayed transiently or in a small number of cells [7]. M1 macrophages/microglia secrete proinflammatory cytokines and upregulate iNOS and reactive oxygen species, which are both neurotoxic and prevent axonal regeneration [2, 7, 27, 37]. The environment of the spinal cord in the later stages of SCI switches macrophages/microglia to the M2 phenotype, thereby releasing trophic factors (e.g., insulin-like growth factor, brain derived neurotrophic factor, and nerve growth factor), and anti-inflammatory cytokines (e.g., IL-4, IL-10, and TGF-β), inducing neuroprotection and promoting axonal regeneration [1, 34]. Whether the mechanism underlying these switches involves the recruitment of circulating precursors or the re-education of cells *in situ* remains unclear [27]. In this study, LPS + IFN-γ treatment of cultured macrophages/microglia from PD-1 deficient mice promoted M1 polarization of these cells, and increased their expression of iNOS and proinflammatory cytokines. In contrast, IL-4 reduced M2 polarization of these cells from PD-1 deficient mice by decreasing their expression of Arg1 and CD206. We also showed that, under *in vivo* conditions, PD-1 deficiency delayed the switch from the M1 to M2 phenotype at the injured site and exacerbated locomotor recovery after SCI. At the early stages of SCI in WT mice, infiltrating macrophages and resident microglia are polarized towards the M1 phenotype with induced expression of PD-1 in the proinflammatory milieu [1, 35, 36]. After SCI, macrophages/microglia highly expressing PD-1 recruit PD-L1 from other macrophages/microglia or astrocytes at the lesion site to provide a negative feedback signal [1, 35, 36]. This effect decreases the high expression of iNOS and proinflammatory cytokines, and also skews the polarization of macrophages/microglia towards the M2 phenotype [23, 13, 16, 32].

Canonical IFN regulatory factor/STAT signaling pathways activated by IFN and Toll-like receptor (TLR) signaling promote the formation of M1 macrophages via STAT1 activation [3, 27]. In contrast, IL-4 and IL-13 promote the M2 phenotype via STAT6 activation [3, 27]. Blocking IFN-γ- and IL-4-mediated STAT1 activation for T helper cell 1 polarization and STAT6 activation for T helper cell 2 polarization, respectively, has been well established [38]. A similar mechanism may underlie the exclusivity of M1 and M2 phenotypes in macrophages [3]. Upon activation of PD-1 by PD-L1, SHP-2 or SHP-1/SHP-2 is recruited to bind to its cytoplasmic domain ITIM and immunoreceptor tyrosine-based switch motif [11]. SHP-2 has been shown to inhibit IFN-γ stimulation of the JAK-STAT signaling pathway [39]. Electrophoretic mobility shift assay and site-directed mutagenesis have shown that IFN-sensitive responsive element, STAT1, and STAT2 are primarily responsible for the constitutive expression of PD-1, as well as for the IFN-α-mediated upregulation of PD-1 [14]. Furthermore, the cross-talk between PD-1 and the suppressor of cytokine signaling-1 negatively regulates IL-12 expression by limiting STAT1 phosphorylation in monocytes/macrophages from hepatitis C virus infection [16, 32]. In this study, STAT1 phosphorylation was enhanced in PD-1-KO macrophages/microglial cells treated with LPS + IFN-γ, thus promoting the M1 phenotype. Decreased STAT6 phosphorylation of macrophages/microglial cells in the presence of IL-4 thereby polarized these cells toward the M2 phenotype. Increased levels of p-STAT1 in the injured spinal cord were also confirmed in PD-1-KO mice after SCI. However, the cross-talk between PD-1 signaling and other signaling pathways of cytokines, particularly IFN-γ and IL-4, is not well understood, and needs to be further investigated in the future.

LPS-activated macrophages are often classified as M1 macrophages owing to their increased levels of iNOS and IL-12, and to the fact that TLR4 activation leads to NF-κB activation [3, 27]. SHP-1 recruits the phosphorylation of ITIM domains on inhibitory receptors. Studies by An et al. [40] have shown that SHP-1 negatively regulates TLR-mediated production of proinflammatory cytokines by inhibiting the activation of NF-κB and mitogen-activated protein kinase. PD-1 has also been shown to inhibit LPS-mediated IL-12 production and differentiation in murine macrophage RAW264.7 cells [13]. In this study, PD-1 deficiency increased NF-κB activation after SCI. However, more studies are needed to demonstrate that PD-1 directly regulates macrophage/microglial polarization via NF-κB.

Another important aspect of the functional polarization of macrophages is phagocytosis [28]. Previous studies have shown that IL-4 and IL-13 enhance fluid-phase pinocytosis and mannose receptor-mediated uptake by the activation of phosphatidylinositol 3-kinase. In contrast, IL-10 or IFN-γ decreased both fluid phase pinocytosis and mannose receptor-mediated uptake [41]. In this study, we found remarkable differences between polarized macrophages and microglial phagocytosis in culture. M2 macrophages derived from WT mice displayed higher phagocytic ability than M1 macrophages. However, the M2 phenotype in WT microglial cells displayed a lower phagocytic ability than M1 cells. This is in agreement with a previous report, which showed that M2 macrophages phagocytize rituximab-opsonized leukemic targets more efficiently than M1 cells in culture [42]. Our findings suggest that PD-1 deficiency causes a divergence of phagocytosis in macrophages and microglia. In WT mice, PD-1 deficiency decreased and increased the phagocytic ability of macrophages and microglia, respectively, and retained the characteristic differences between the M1 and M2 phenotypes. Microglial cells originate from the yolk sac and populate the CNS early in development; moreover, these cells have an extended life span with little turnover [43]. Myeloid cells of hematopoietic origin populate the perivascular space and have a relatively rapid rate of repopulation. Under inflammatory conditions and injury, hematogenous myeloid cells (macrophages) access the CNS parenchyma. Currently, there are no distinct lineage markers that distinguish microglia from infiltrating macrophages [2]. In this study, PD-1 deficiency promoted different regulatory roles in phagocytosis, suggesting that significant heterogeneity exists between macrophages and microglia. In this study, microglial cells were derived from neonatal animals and macrophages were derived from adult animals. Phagocytosis of pathogenic bacteria and charcoal particles is impaired in aged microglia and macrophages [44]. In view of these findings, the different ages of these cultured cells in our study may have resulted in their differing roles of phagocytosis in conditions of PD-1 deficiency. More work is required to explore the exact mechanism.

Regulation of the immune response is a promising strategy for successful SCI repair, and macrophages are considered prospective candidates for cell therapy [45]. This study provides new insights into the modulatory mechanisms of macrophage/microglial polarization by shedding light on new therapies for SCI via PD-1-mediated polarization of macrophages/microglia.

## Electronic supplementary material

Below is the link to the electronic supplementary material.

Supplementary Fig. S1 Expression of the ligand of programmed death-1 (PD-L1) in astrocytes after spinal cord injury in mice. Immunohistochemistry for glial fibrillary acidic protein (GFAP; astrocytes) 14 days postinjury in wild-type mice. (+) Injury epicenter. Scale bar = 100 μm (GIF 52 kb)

High resolution image (TIFF 7282 kb)

ESM 1 (PDF 603 kb)

ESM 2 (PDF 612 kb)
