# Supplementary figures and images for: Programmed Death 1 Deficiency Induces the Polarization of Macrophages/Microglia to the M1 Phenotype After Spinal Cord Injury in Mice
Source: Neurotherapeutics. 2014 May 23;11(3):636–50. doi: 10.1007/s13311-013-0254-x (PMC4121443; doi:10.1007/s13311-013-0254-x)

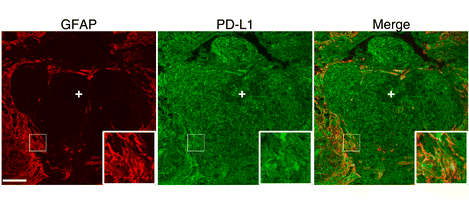

Supplement: Supplementary file 1 — Expression of the ligand of programmed death-1 (PD-L1) in astrocytes after spinal cord injury in mice. Immunohistochemistry for glial fibrillary acidic protein (GFAP; astrocytes) 14 days postinjury in wild-type mice. (+) Injury epicenter. Scale bar = 100 μm (GIF 52 kb) [file 13311_2013_254_Fig8_ESM.gif]

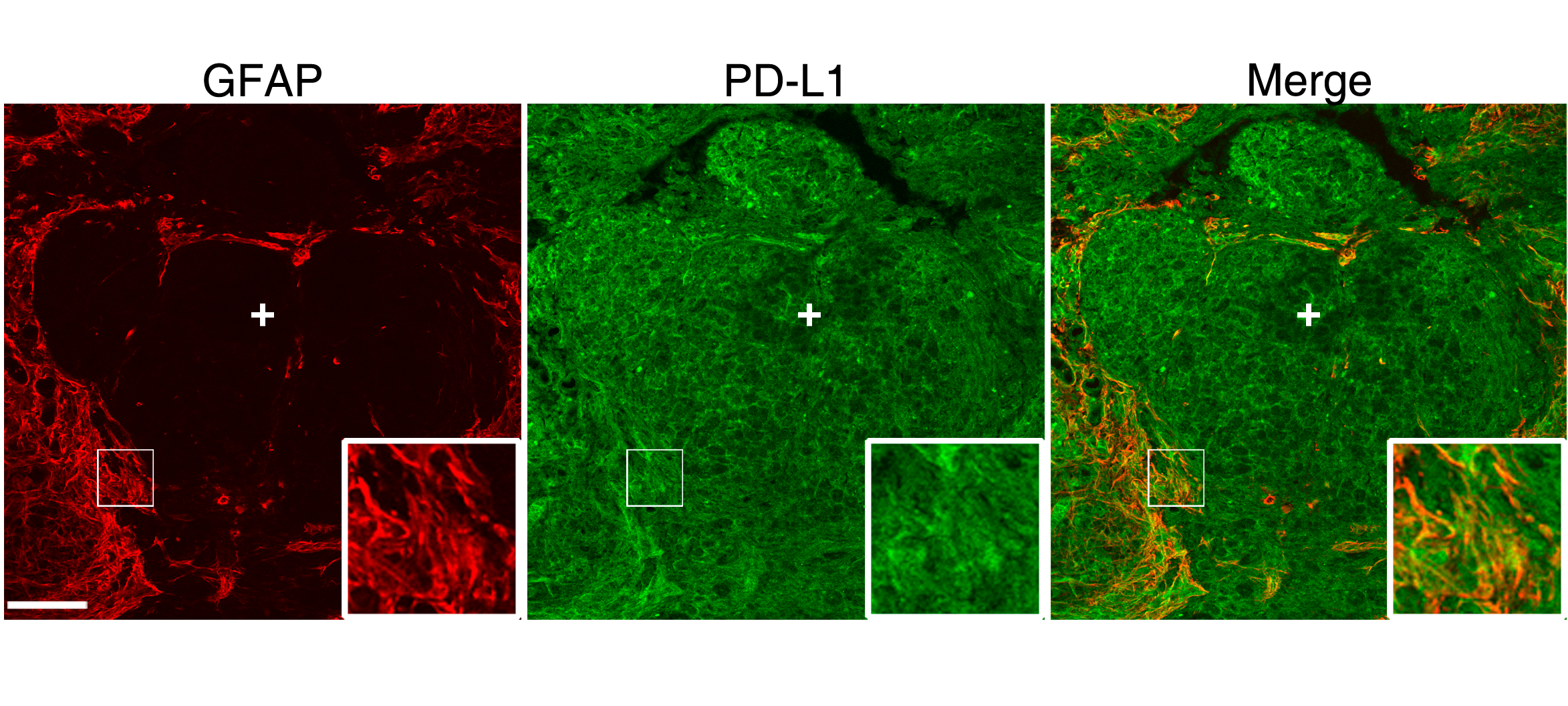

Supplement: Supplementary file 2 — High resolution image (TIFF 7282 kb) [file 13311_2013_254_MOESM1_ESM.tif]
